# The Effects of Boric Acid Sugar Bait on *Wolbachia* Trans-Infected Male *Aedes albopictus* (ZAP Males^®^) in Laboratory Conditions

**DOI:** 10.3390/insects13010001

**Published:** 2021-12-21

**Authors:** Vindhya S. Aryaprema, Whitney A. Qualls, Karen L. Dobson, Stephen L. Dobson, Rui-De Xue

**Affiliations:** 1 Anastasia Mosquito Control District, 120 EOC Drive, St. Augustine, FL 32092, USA; varyaprema@amcdfl.org (V.S.A.); wqualls@amcdfl.org (W.A.Q.); 2 MosquitoMate, Inc., Lexington, KY 40503, USA; kdobson@mosquitomate.com (K.L.D.); sdobson@mosquitomate.com (S.L.D.); 3 Department of Entomology, University of Kentucky, Lexington, KY 40546, USA

**Keywords:** *Wolbachia*, toxic sugar baits, *Aedes albopictus*, mosquito control

## Abstract

**Simple Summary:**

The release of *Wolbachia* trans-infected mosquitoes to the environment has demonstrated promising results in reducing the target mosquito populations. The use of boric acid toxic sugar bait is another successful and upcoming technique in mosquito control. However, the potential complementary use of the two techniques had not been evaluated. This study demonstrates the significant mortality impact of boric acid toxic sugar bait on *Wolbachia* trans-infected *Aedes albopictus* Skuse mosquitoes, thus giving important insight to program planners.

**Abstract:**

The field release of *Wolbachia* trans-infected male mosquitoes, as well as the use of toxic sugar baits, is a novel and promising candidate technique for integrated mosquito management programs. However, the methods of action of the two techniques may not be complementary, because the *Wolbachia* method releases mosquitoes into the environment expecting a wild population reduction in subsequent generations while the toxic baits are intended to reduce the wild population by killing mosquitoes. This laboratory study was conducted to evaluate the effectiveness of boric acid toxic sugar baits on *Wolbachia* trans-infected male *Aedes albopictus*, relative to wild-type *Ae. albopictus* males. *Wolbachia* trans-infected (ZAP male^®^) and the wild-type *Ae. albopictus* males were exposed separately to 1% boric acid in a 10% sucrose solution in BugDorms. In the control test, the two groups were exposed to 10% sucrose solution without boric acid. Percent mortalities were counted for 24 h, 48 h and 72 h post exposure periods. The results show that 1% boric acid toxic sugar bait can effectively kill ZAP males under laboratory conditions, and the effectiveness was significantly higher after 24 h and 48 h, compared to wild-type male *Ae. albopictus*. This finding will help in planning and coordinating integrated mosquito management programs, including both *Wolbachia* trans-infected mosquito releases and the use of toxic sugar baits against *Ae. albopictus*.

## 1. Introduction

Historically, the application of synthetic insecticides has been the gold standard for mosquito control. However, with the development and spread of insecticide resistance, mosquito control has become more challenging, which has created a demand for novel techniques such as sterile insect techniques (SIT) [1]. The use of an intracellular bacterium *Wolbachia* to reduce *Aedes* mosquito populations is one of the promising techniques [1]. *Wolbachia pipientis* is a naturally occurring intracellular bacterium that is commonly found in most insect species, including mosquitoes. Different strains of *Wolbachia* are present in different species. *Wolbachia* are maternally inherited [2] and the infection is passed to the next generation via the embryonic cytoplasm [1]. *Wolbachia* infections cause the embryonic death of mosquitoes through cytoplasmic incompatibility (CI) resulting from sperm–egg incompatibility [2]. This CI occurs when *Wolbachia*-infected male mosquitoes mate with female mosquitoes that are not infected or are infected with a different *Wolbachia* strain [3]. This phenomenon has been used successfully in mosquito control for population suppression [4], as well as for population replacement [5]. Population suppression is achieved by releasing only *Wolbachia*-infected male mosquitoes of the target species into the environment, while population replacement is achieved by releasing both sexes of mosquitoes infected with the same *Wolbachia* strain. Population replacement is used mainly to control disease transmission, with *Wolbachia* strains that can interfere with the transmission of pathogens such as the dengue virus [4]. Both population suppression and population replacement require releases of large numbers of *Wolbachia* trans-infected mosquitoes into the target environment [3,6,7].

*Aedes albopictus* Skuse, a nuisance [8] and a vector mosquito of many diseases in the world [9], is naturally superinfected with *w*AlbA and/or *w*AlbB strains of *Wolbachia* [10]. A new strain of *Ae. albopictus* has been developed by the MosquitoMate^®^, Inc., Lexington, KY, by trans-infecting this superinfected *Ae. albopictus* with the *w*Pip strain of *Wolbachia,* which occurs naturally in *Culex pipiens* complex [11]. The release of these trans-infected *Ae. albopictus* male mosquitoes into the environment causes CI in the wild population, resulting in population suppression in the subsequent generations [12].

The use of toxic sugar baits (TSBs) is another novel technique that targets the sugar-feeding behavior of mosquitoes. TSBs are used as bait stations or barrier sprays and have been successfully evaluated for the control of mosquito populations [13,14,15,16]. Boric acid as toxic sugar bait has demonstrated effective control for adult mosquitoes [17].

Unlike the *Wolbachia* trans-infection technique, TSB technique is not species-specific and is used for the control of multiple species of adult mosquitoes. Therefore, the two techniques could be possible candidates for integrated mosquito management (IMM) programs. However, unlike the gradual decline of populations by the *Wolbachia* population suppression technique, TSBs had an immediate impact by killing the individuals that fed on it. The effects of fitness costs of the *Wolbachia* trans-infections of mosquitoes were not totally explored, and there is no evidence on the impact of chemical control, including TSBs, on released *Wolbachia* trans-infected mosquitoes. Also, during the mass release of SIT male mosquitoes in a large residential area, there are a few residential yards that had received the applications of TSB stations or barrier treatments by local pest-control companies. So far, we do not know whether the TSB applications impact the efficacy of released SIT mosquitoes, including the release of *Wolbachia* trans-infected mosquitoes. This study was carried out to determine whether the boric acid toxic sugar baits impact *Wolbachia* trans-infected male *Ae. albopictus* (ZAP hereafter). Understanding the effects will benefit the planning and coordinating effective IMM programs that combine the two techniques appropriately to control different mosquito species.

## 2. Materials and Methods

Similarly aged ZAP and wild-type *Ae. albopictus* males (Wild) were received from MosquitoMate (24 h old when shipped and received 24 h later). The age of mosquitoes to be tested was selected based on the fact that ZAP mosquitoes are usually released into the environment ~48 h after emergence. A total of 12 BugDorms (30 cm × 30 cm × 30 cm) (Bioquip, Rancho Dominguez, CA, USA) were used in the study, each having 100–150 mosquitoes of either Wild or ZAP mosquitoes.

Mosquitoes were allowed to acclimatize to the environment for one and a half hours. The experiment was set up with two lines, treatment and control lines. In the control line, three cages of each group were provided with a blue-dyed (Blue No. 1 FD & C Dye, Ingredient Depot, Amazon.com) 10% sugar solution. In the treatment line, three cages of each group (i.e., ZAP and Wild mosquitoes) received a blue-dyed 10% sugar + 1% boric acid solution as the TSB. All the cages were kept in an insectary at temperature 26 °C ± 2, relative humidity (70–80%) and 14L:10D photoperiod. Dead mosquitoes were removed, and numbers of dyed dead mosquitoes in all cages were recorded at 24, 48 and 72 h post exposure periods. Mosquitoes were recorded as dead if they were unresponsive to a gentle touch of the aspirator or forceps. Dyed dead mosquitoes were considered dead due to the treatment and were identified by the blue dye patches when squashing them on a white paper. If not visually conspicuous, squashed mosquitoes were observed under a microscope for any blue dye. The total number of exposed mosquitoes were determined from the cumulative total number of dyed dead mosquitoes and those left alive after 72 h. Percent mortalities were calculated for each period and for each cage. The same experiment was replicated three times over three weeks. Whenever the control mortalities were between 5–10% in any replicate, the corresponding treatment mortalities were corrected using Abbott’s formula [18] to eliminate effects on mortality by any confounding factors other than the treatment.

A generalized linear model (GLM) was used for a Poisson distributed dependent variable of the percent cumulative mortality of mosquitoes. Independent variables in the model are *Wolbachia* infection (yes-ZAP/no-Wild), group (control/treatment), exposure time (24 h, 48 h, 72 h), and replication (1, 2, 3). Besides the main effects, the model also included a 3-way interaction for *Wolbachia* infection, group, and time. Planned comparisons were made for treatment vs. control within each *Wolbachia* infection (i.e., ZAP and Wild), at each post-exposure time. A second set of planned comparisons were made between *Wolbachia* infections for treatment groups at each exposure time, and a third set of planned comparisons were made between *Wolbachia* infections for control groups at each exposure period. Comparison *p*-values of 0.05 or less were considered to be statistically significant.

## 3. Results

Both Wild and ZAP *Ae. albopictus* groups showed >95% mortality after 72 h exposure to the TSB. Mortalities of corresponding control groups were between 3.4–5.1% (Wild and ZAP, respectively) (Table 1). Treatment mortalities of both Wild and ZAP groups were significantly higher at all three post-exposure periods compared to the corresponding control mortalities (Table 1). The cumulative mortalities of both treatment and control mosquitoes of the Wild group were significantly increased with the increasing post-exposure period (Table 1 and Table 2). Similarly, the control cumulative mortalities of the ZAP group were significantly increased with the increasing post-exposure period, whereas the treatment cumulative mortality was significantly higher at 48 h exposure periods than 24 h but not between 48 h and 72 h. The highest ZAP treatment mortality was at 72 h post-exposure without significant difference between 48 h (94.4%) and 72 h (99.5%) post-exposure (Table 1 and Table 2). At the same time, the Wild group had only 77.8% mortality at 48 h and the highest mortality of 97% at 72 h.

The mortality of the treated ZAP group was significantly higher than that of the treated Wild group at 24 h and 48 h post-exposure but not at 72 h. (Table 1 and Table 3). Similarly, significantly higher mortalities were observed in the control ZAP group compared to the control Wild group at 24 h and 48 h exposure but not at 72 h (Table 1 and Table 3).

## 4. Discussion

The results indicate that 1% boric acid toxic sugar bait is highly effective in killing off (>95% mortality) both wild-type (Wild) and *Wolbachia* trans-infected (ZAP) male *Ae. albopictus* within a 72 h exposure period under laboratory conditions. Although the level of effectiveness was low at lower exposure periods, the effectiveness of the TSB against Wild as well as ZAP groups of *Ae. albopictus* was significantly higher at each exposure period compared to controls. At the same time, TSB had incurred a higher and a faster mortality in the ZAP group, with 94% mortality at 48 h post-exposure, compared to 78% mortality in the Wild group at the same time point. This may be due to the reported increase in the metabolism of *Wolbachia*-infected mosquitoes [19], which may have caused more frequent sugar feeding. The trans-infection of an additional *Wolbachia* strain (*w*Pip) to a naturally superinfected *Ae. albopictus* might have caused a higher metabolism in the ZAP group than in the Wild group. Higher mortalities in both the control and treated mosquitoes of the ZAP group at 24 h and 48 h exposure periods, compared to the Wild group, might be attributed to fitness costs, as different *Wolbachia*-infected mosquito strains vary in their relative fitness impacts [20,21,22]. The increasing natural death rate of mosquitoes and/or mortality due to other confounding factors with time might have contributed to the non-significant differences in mortality between the Wild and Zap groups of both control and treatment mosquitoes. The study demonstrated the high effectiveness of 1% boric acid toxic sugar bait against *Wolbachia* trans-infected *Ae. albopictus* male mosquitoes within 48 h, which was higher and faster against wild-type *Ae. albopictus* under laboratory conditions. It would reduce the released mosquito numbers by >90% in 48 h and almost 100% in 72 h. However, the *Wolbachia* technique requires the released male mosquitoes to disperse well in the environment and compete with wild males to find their mates. The mating success of released males should be sufficiently large to overcome the natural rate of population increase. Therefore, the two novel mosquito control techniques, the release of *Wolbachia* trans-infected *Ae. albopictus* which is species-specific and the use of non-species-specific 1% boric acid toxic sugar bait, would not be complementary in the temporal dimensions of IMM programs and need to be well planned and coordinated. In addition, the status of the application of TSBs in the planned/targeted area should be checked, at least a survey about the TSBs or other control methods should be conducted before the release application of ZAP mosquitoes or other SIT mosquitoes. Using TSB technique first to reduce natural populations of multiple species in the target area and then release *Wolbachia* trans-infected mosquitoes to eliminate/minimize the remaining population of the target species would be more effective and faster against wild-type mosquitoes. Further investigations in natural environments will help confirm these laboratory findings so that IMM programs that desire to combine the *Wolbachia* and TSB techniques to control populations of different mosquito species will be planned and coordinated appropriately to obtain the maximum benefit.

## Figures and Tables

**Table 1:** Comparison of percent cumulative mortality between treatment (T) and control (C) groups of wild-type (Wild) and *Wolbachia* trans-infected (ZAP) *Aedes albopictus* males (treatment group exposed to 1% boric acid toxic sugar bait for different exposure periods, SE = standard error of the mean).

	Wild				ZAP			
	Mean (SE)		t-Value (df = 86)	*p*-Value	Mean (SE)		t-Value (df = 86)	*p*-Value
	C	T			C	T		
24 h	0.35 (0.19)	15.57 (1.31)	−7.04	<0.0001	1.61 (0.41)	26.91 (1.73)	−10.63	<0.0001
48 h	1.44 (0.38)	77.81 (2.94)	−14.87	<0.0001	3.26 (0.60)	94.44 (3.24)	−18.21	<0.0001
72 h	3.38 (0.60)	96.98 (3.28)	−18.65	<0.0001	5.14 (0.75)	99.57 (3.33)	−19.80	<0.0001

**Table 2:** Comparison of percent cumulative mortality between different exposure periods of the control and treatment groups of wild-type (Wild) and *Wolbachia* trans-infected (ZAP) *Aedes albopictus* males (treatment group exposed to 1% boric acid toxic sugar bait).

	Wild				ZAP			
	Control		Treatment		Control		Treatment	
	t-Value (df = 86)	*p*-Value	t-Value (df = 86)	*p*-Value	t-Value (df = 86)	*p*-Value	t-Value (df = 86)	*p*-Value
24 h/48 h	−2.39	0.02	−17.4	<0.0001	−2.27	0.03	−17.2	<0.0001
48 h/72 h	−2.72	0.008	−4.34	<0.0001	−1.98	0.05	−1.1	0.27

**Table 3:** Comparison of percent cumulative mortality between wild-type (Wild) and *Wolbachia* trans-infected (ZAP) *Aedes albopictus* males of treatment and control groups (treatment group exposed to 1% boric acid toxic sugar bait).

	Treatment		Control	
	t-Value (df = 86)	*p*-Value	t-Value (df = 86)	*p*-Value
24 h	−5.18	<0.0001	−2.59	0.01
48 h	−3.78	0.0003	−2.58	0.01
72 h	−0.55	0.58	−1.86	0.07

## Data Availability

Not applicable.
